# Epidemic Cholera

**Published:** 1849-02

**Authors:** 


*Epidemic Cholera.*— Supposing that our readers are desirous of being advised of facts pertaining to the progress of this pestilence in different parts of the world, we shall continue to present such information as we can gather from authentic accounts within our reach. The following is an extract contained in an Edinburg monthly Med. Journal, and copied by the Philadelphia Med. News for January.

The epidemic which we announced in our last month's publication as having invaded this country, has continued to rage with great virulence in Edinburg, where the number of cases now amounts to between seven and eight hundred (including Leith,) of which nearly a half have already proved fatal, about 230 remaining under treatment. A severe epidemic broke out at Loanhead (a village six miles to the south of Edinburg,) and many cases have also occured at Lasswade, Gilmerton, Portobello, Gockpen, and other villages of the neighborhood. Several cases have been reported in Glasgow, mostly in very crowded and miserable localities; and the disease is also reported to exist in Dumfries as well as in Falkirk, a town whose sanatory condition is far from satisfactory. In England the number of cases has not approached that of the northern division of the kingdom; and, although in London the number has been considerable, yet relatively to the population, it is very small compared with Edinburg. On the week ending October 4th, 65 deaths were recorded in the Registrar General's returns from Cholera in London: on November 11th, 62; on November 18th 54. The epidemic appears, therefore, on the whole, to be decreasing in violence in London. Everywhere the disease appears to have been almost confined to the most wretched and miserable of the population; a few trivial exceptions only to this rule having occurred in London and Edinburg. The mortality has been everywhere nearly the same,—viz., about two-thirds of the recorded cases which have been brought to a termination. It is probable, however, that no trustworthy returns of the whole cases in any place have been yet published.

On the continent it has declined in a great measure at St. Petersburg, Berlin, and Hamburg, where, however cases still occur. On the other hand, Rotterdam has been attacked with great severity since the 1st of October, and Dantzic, from a similar date, has suffered under a still more severe visitation. It is stated that in the small town of Gartz, in the district of Settin, there has been the extraordinary mortality of 102 persons out of a population of 700.

A considerable number of cases of a choleroid disease prevailed in the beginning of the month in Dunkirk; and a few have occurred in the neighboring villages, and in Calais. M. Magendie was sent by the Academy of Sciences to investigate this alleged ingress of the disease into France; and reported the cases not to be true epidemic cholera. If we may trust the report of a discussion in the Société de Médicine Pratique, several cases having the strongest resemblance to the disease appear to have occurred in Paris; but there seems to be, on the part of the French physicians, a strong disposition to doubt or deny its presence.

In another portion of the article above referred to, it is stated, that chloroform has been tried assiduously in hospital practice in Edinburgh, but without the least success as regards cure. It alleviates the spasms and other painful symptoms.

The *London Med. Gazette, Nov.* 24, states, that the total number of cases reported, in London, up to Nov. 22nd, to be 396. Number of deaths 212. This exhibits a very high rate of mortality.

"The total number of cases in England (Boston Med. and Surg. Jour.) up to Dec. 20, amounted to 3737, whereof 1772 had proved fatal; 505 had recovered; and 1400 result not recorded, or still under treatment, The cases in Scotland have been no fewer than 2922, whereof 1356 have perished."

The *Western Lancet* for Jan., states that four cases of cholera have been received at the Commercial Hospital, at Cincinnati. The epidemic has made its appearance at several places on the Mississippi and Ohio rivers.

